# Early-life exposure to bisphenol A and reproductive-related outcomes in rodent models: a systematic review and meta-analysis

**DOI:** 10.18632/aging.103620

**Published:** 2020-09-30

**Authors:** Xiaohan Ren, Tongtong Zhang, Xinglin Chen, Xiyi Wei, Ye Tian, Guangyao Li, Xu Zhang, Wei Zhang, Zebing You, Shangqian Wang, Chao Qin

**Affiliations:** 1 The State Key Laboratory of Reproductive Medicine, Department of Urology, The First Affiliated Hospital of Nanjing Medical University, Nanjing 210029, China

**Keywords:** bisphenol A, sperm, reproductive, meta-analysis

## Abstract

We performed this meta-analysis to elucidate the associations between early-life BPA exposure and reproductive-related outcome indicators. The standardized mean differences (SMDs) and its 95% confidence intervals (CIs) were measured by fixed-effects or random-effects models. The results revealed that BPA exposure at extremely-high dose (>50mg/kg/day) was significantly associated with negative reproductive-related outcomes (Prostate weight: SMD: -4.21; 95% Cl: -5.97, -2.44; Testis weight: SMD: -1.92; 95% Cl: -2.61, -1.23; Epididymis weight: SMD: -2.16; 95% Cl: -3.47, -0.86; Daily sperm production; SMD: -1.90; 95% Cl: -3.27, -0.53; Epididymal sperm count; SMD: -3.42; 95% Cl: -3.87, -2.97). Meanwhile, regardless of the dose, early-life BPA exposure could result in an adverse effect on sperm parameters of F1 generation male rodents at any period. Also, we found the non-monotonic dose response curves of BPA in specific tissues or organs, which may challenge the traditional mindset of "safe dose". This study demonstrated that bisphenol A exposure was relevant to adverse reproductive-related outcomes at specially appointed dose and period of life. Yet the assumption that no adverse effects can occur below the "safe" dose is suspected.

## INTRODUCTION

Bisphenol A (BPA) is a high-production-volume chemical extensively used in the production of plastics, epoxy resin linings of food packaging, coatings, and fillings, and now leaches into the environment with over one million pounds each year [1]. Despite its low affinity, BPA could bind classical nuclear estrogen receptor (ER) alpha and beta, as well as membrane-associated GPR30, and may inhibit the function of ER and other nuclear hormone receptors for its similar structure as diethylstilbestrol (DES), a highly potent ER agonist [2].

Owing to the ubiquitous presence of BPA in the environment and its serious endocrine-disrupting effects, coupled with the increased incidence of endocrine-associated cancer, many studies have evaluated the carcinogenesis of BPA in hormone-sensitive organs (prostate, testis, breast and ovary et al.) [3, 4]. The US Food and Drug Administration (FDA) has set the safe reference dose (RfD) at 50 μg/kg/d for humans based on a 1,000-fold reduction of the dose used in the rodent study [5]. Recently, the European Food Safety Authority (EFSA) lowered the RfD to 4 μg/kg/d after reevaluating the toxicological data for BPA [6].

To explore the role of BPA on the reproductive system, scholars have carried out many experiments based on rodent models and achieved preliminary results. However, due to the differences in animal models, dosage, administration methods, and the conflicts of interest behind the experiment, the results of the experiments were controversial. For example, a study sponsored by the Polycarbonate/BPA Global Group, which is an organization promoting the welfare and interests of polycarbonate plastics and BPA manufacturers, stated that, in the SD (Sprague-Dawley) rats model, the cancer risk of any organ system exposed to BPA for a long time did not increase regardless of the exposure dose and period [7]. However, another study evaluating BPA exposure prenatally administered to SD rats suggested that at postnatal day (PND) 180, low dose BPA exposure was sufficient to induce hyperplasia/dysplasia of the prostate (control group: 0% vs. BPA group: 62%) [8].

We performed this study to provide a more rigorous assessment of the existing rodent model by systematically reviewing experimental studies reporting BPA and detrimental outcomes. We reviewed and conducted a meta-analysis of these rodent studies which reported the relationship between prenatal or perinatal (early-life) BPA exposure and the following reproductive-related outcomes: body weight, prostate weight, testis weight, epididymis weight, seminal vesicle weight, sperm motility, daily sperm production (DSP; testis), efficiency of sperm production, and epididymal sperm concentration (Table 1).

**Table 1:** PECO statement (population, exposure, comparator and outcomes).

**Variable**	**Description**
Population	Experimental rodent studies
Exposure	Early-life exposure to bisphenol A (prenatal and early postnatal; have a exposure history during gestation period to postnatal day 21)
Comparator	Animals exposed to vehicle-only treatment
Outcomes	body weight, prostate weight, testis weight, epididymis weight, seminal vesicle weight, sperm motility, daily sperm production (testis), efficiency of sperm production (g/testis/day), and epididymal sperm concentrations

## RESULTS

### Characteristics of included studies

**Figure 1:**
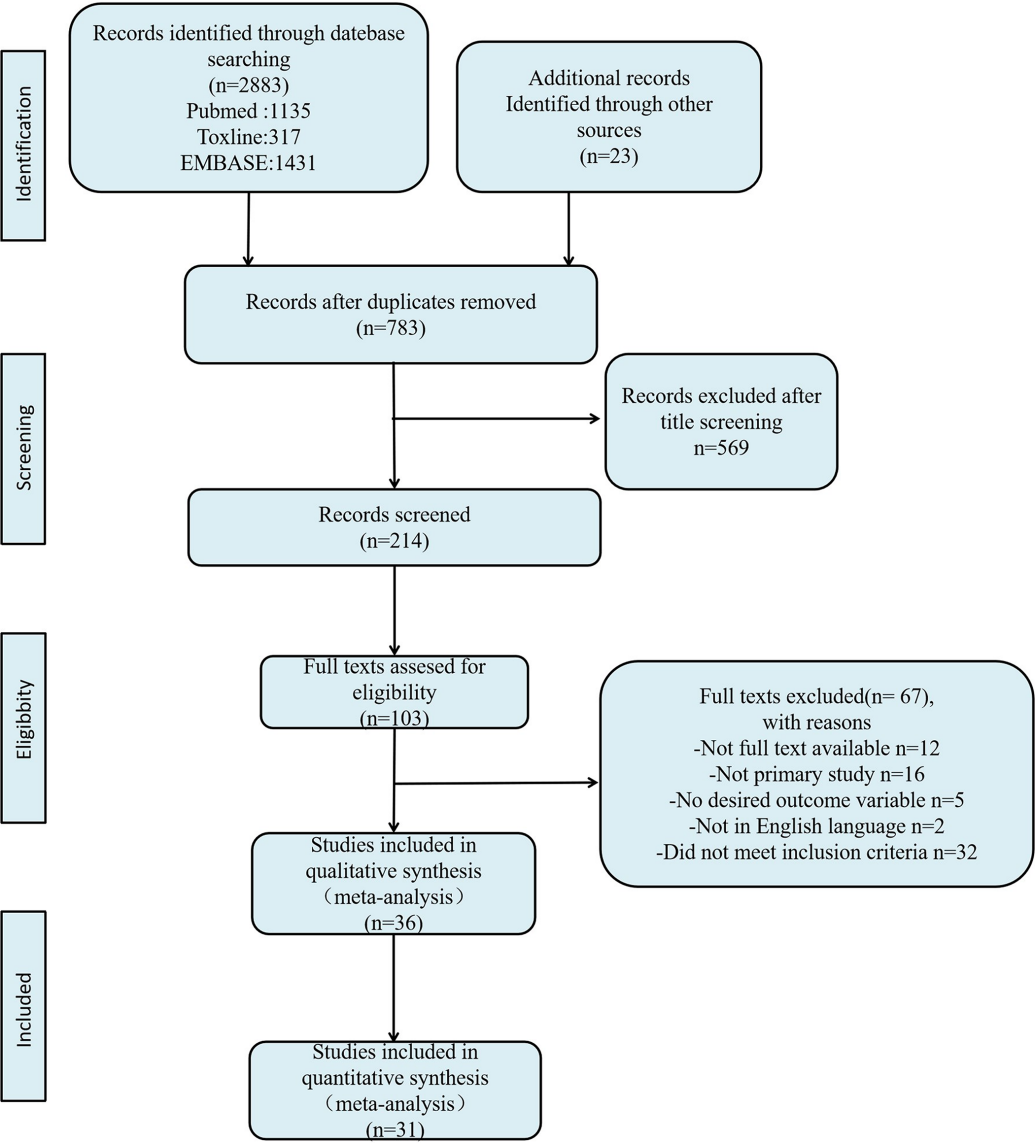
**Flow diagram of literature search and selection process.**

The flowchart of the literature search and selection process is shown in Figure 1. After searching PubMed, EMBASE, Toxline databases, and additional references for relevant articles, a total of 2883 articles were found. Of these articles, 103 articles met the criteria for full-text review. Eventually, 31 articles were included for analysis (Table 2) [7, 8, 16–44].

**Table 2:** Characteristics of the studies included in the meta-analysis.

**Reference**	**Year**	**Species (strain)**	**Dose in μg/kg bw/day**	**Exposure period**	**Exposure route**	**Timing of outcome measurement**	**Effects on male reproduction extracted in the analysis**
Dabeer et al. [16]	2020	Rat (Wistar)	10000	180 days prebreed-PND35	Drinking water	PND35	Organ weight, Sperm parameters
Shi et al. [17]	2018	Mice (CD-1)	0.5/20/50	GD11-PND1	Oral	PND60, PND120	Body weight, Organ weight, Sperm parameters
Prints et al. [18]	2018	Rat (Sprague-Dawley)	2.5/25/250/2500/ 25000	Grp1:GD6-PND365; Grp2:GD6- PND21	Gavage	PND365	Body weight
Spörndly-Nees et al. [19]	2018	Rat (F344)	0.5/50	GD3.5-GD22	Drinking water	PND35, PND360	Body weight, Organ weight
Dere et al. [20]	2018	Rat (Sprague-Dawley)	2.5/25/250/2500/25000/250000	GD6-PND90	Gavage	PND90	Body weight, Organ weight
Rahman et al. [21]	2017	Mice (CD-1)	50/5000/50000	GD7-GD14	Gavage	PND120	Sperm parameters
Bernardo et al. [22]	2015	Rat (Sprague-Dawley)	25/250	GD10-GD21	Gavage	PND21, PND180	Body weight, Organ weight
Rodrıguez et al. [23]	2015	Gerbil	40	GD17-24	Oral	PND1, PND90	Body weight, Organ weight
Hass et al. [24]	2015	Rat (Wistar)	25/250/5000/50000	GD7-PND22	Gavage	PND90	Body weight, Organ weight
Delclos et al. [25]	2014	Rat (Sprague-Dawley)	2.5/8/25/80/260/840/2700/100000/300000	GD6-PND90	Diet	PND90	Organ weight
Brandt et al. [8]	2014	Rat (Sprague-Dawley)	25/250	GD10-GD21	Gavage	PND21, PND180	Body weight, Organ weight
Gámez et al. [26]	2014	Rat (Wistar)	3	GD1-PND21	Drinking water	PND35	Body weight, Organ weight
Vilela et al. [27]	2013	Mice (Vesper)	40/80/200	GD5-PND1	Gavage	PND70	Sperm parameters
Kendig et al. [28]	2012	Mice (CD-1)	30/300/3000/30000/300000	GD1-PND90	Oral	PND90	Organ weight, Sperm parameters
Kobayashi et al. [29]	2012	Rat (Sprague-Dawley)	330/3300/33000	GD6-PND21	Diet	PND35, PND90	Body weight, Organ weight, Sperm parameters
LaRocca et al. [30]	2011	Mice (C57/Bl6)	50/1000	GD10-GD16	Gavage	PND56	Body weight, Organ weight
Salian et al. [31]	2009a	Rat (Holtzman)	1.2/2.4	GD12-PND21	Gavage	PND125	Body weight, Organ weight, Sperm parameters
Salian et al. [32]	2009b	Rat (Holtzman)	100/200/400/830/1660	PND1-PND5	Injection	PND135	Sperm parameters
Tyl et al. [33]	2007	Mice (CD-1)	3/30/300/5000/50000/600000	40 days prebreed-PND140	Diet	PND140	Body weight, Organ weight, Sperm parameters
Howdeshell et al. [34]	2007	Rat (Long Evans)	2/20/200	GD7-PND1	Gavage	PND150	Body weight, Organ weight, Sperm parameters
Kato et al. [35]	2006	Rat (Sprague-Dawley)	2/11/56/277/97000	PND1-PND9	Injection	PND10, PND35, PND150	Body weight, Organ weight, Sperm parameters
Ichihara et al. [36]	2003	Rat (F344)	50/7500/12000	3 weeks prebreed-PND22	Gavage	PND455	Body weight, Organ weight
Tyl et al. [7]	2002	Rat (Sprague-Dawley)	1/20/300/5000/50000/500000	10 weeks prebreed- PND21	Diet	PND161	Body weight, Organ weight, Sperm parameters
Tinwell et al. [37]	2002	Rat (Wistar)	20/100/50000	GD 6-21	Gavage	PND90	Body weight, Organ weight, Sperm parameters
Yoshino et al. [38]	2002	Rat (F344)	7500/12000	3 weeks prebreed-PND21	Gavage	PND90	Body weight, Organ weight, Sperm parameters
Nagao et al. [39]	2002	Mice (C57BL/6N)	2/20/200	GD11-GD18	Diet	PND84	Body weight, Organ weight, Sperm parameters
Ema et al. [40]	2001	Rat (Sprague-Dawley)	0.2/2/20/200	G1-PND21	Diet	PND120	Body weight, Organ weight
Kwon et al. [41]	2000	Rat (Sprague-Dawley)	3200/32000/320000	GD11-PND20	Oral	PND180	Body weight, Organ weight
Cagen et al. [42]	1999	Mice (CF-1)	0.2/20/200	GD 11-17	Oral	PND90	Body weight, Organ weight, Sperm parameters
Ashby et al. [43]	1999	Mice (CF-1)	20-Feb	GD11-17	Oral	PND183-187	Body weight, Organ weight, Sperm parameters
Vom Saal et al. [44]	1998	Mice (CF-1)	20-Feb	GD11-17	Drinking water	PND180	Body weight, Organ weight, Sperm parameters

**Notes:** PND, postnatal day; GD, gestation day.

The dose of BPA in studies ranged from 0.1 ug/kg/day to 600 mg/kg/day, which was divided by four ascending intervals: 0-60ug/kg/day (low); 100-600ug/kg/day (medium); 1-50mg/kg/day (high); >50mg/kg/day (extremely high). For the timing of data collection, outcomes were measured between PND0 and PND455 and divided by three subgroups: <PND60 (puberty and prepuberty); PND60-PND180 (sexual maturity and adulthood); >PND180 (middle-aged and aged). More details are presented in Table 2.

### Rob evaluation

**Figure 2:**
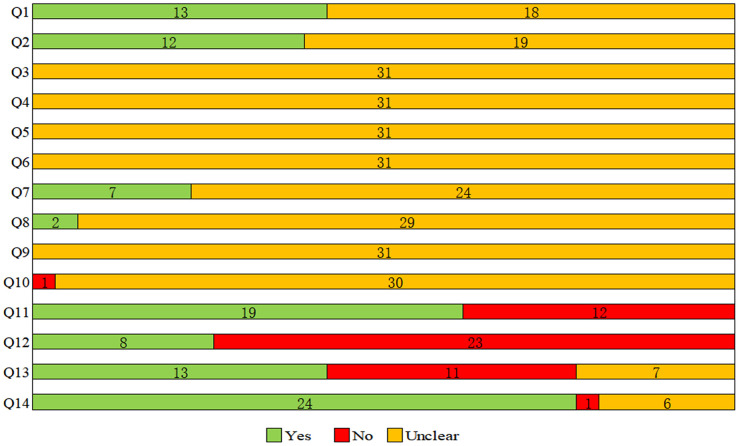
**Results of the risk of bias and methodological quality indicators for all included studies.** Notes: Q, question. Q1: Was the allocation sequence adequately generated and applied?; Q2: Were the groups similar at baseline or were they adjusted for confounders in the analysis?; Q3: Was the allocation to the different groups adequately concealed?; Q4: Were the animals randomly housed during the experiment?; Q5: Were the caregivers and/or investigators blinded from knowledge which intervention each animal received during the experiment?; Q6: Were animals selected at random for outcome assessment?; Q7: Was the outcome assessor blinded?; Q8: Were incomplete outcome data adequately addressed?; Q9: Are reports of the study free of selective outcome reporting?; Q10: Was the study apparently free of other problems that could result in high risk of bias?; Q11: Was it stated that the experiment was randomized at any level?; Q12: Was it stated that the experiment was blinded at any level?; Q13: Was it stated that there was no conflict of interest?; Q14: Was the funding source of the study provided?

The assessment results of Rob and methodological quality were shown in Supplementary Table 2 and Figure 2, with many items receiving an “unclear” rating, resulting in an unknown Rob. For selection bias (Q1-Q3), 13 studies reported the sequence generation process (Q1); 12 studies reported on the adjustment of confounding factors and baseline characteristics of two groups (BPA group and control group) (Q2); none of study reported the allocation concealment (Q3). Regarding performance bias (Q4, Q5), the random housing and blinding of caregivers were not reported at all. On the aspect of detection bias (Q6, Q7), outcome assessor was described blinded in 7 studies (Q7), whereas no study reported the randomization of outcome measure (Q6). For attrition bias (Q8), 2 studies adequately addressed incomplete outcome data. All studies had an unclear risk of reporting bias (Q9). Besides, one study (Nagao 2002) was scored with a high risk of other bias for cesarean section (Q10). With respect of methodological quality (Q11-Q14), 19 studies reported randomization at any level of the experiment (Q11); only 8 studies reported the blinding (Q12); 13 studies stated that there was no conflict of interest (Q13); and the funding source was provided in 25 studies (Q14).

### Effects of BPA on reproductive-related outcomes

The overall meta-analysis results of enrolled studies are shown in Table 3 and Supplementary Table 3. Forest figures are shown in Supplementary Figure 2–10.

**Table 3:** Meta-analysis results of enrolled studies.

	**0-60ug/kg/day**	**Pa**	**100-600ug/kg/day**	**Pa**	**1-50mg/kg/day**	**Pa**	**>50mg/kg/day**	**Pa**
**Body weight**								
<PND60	0.07(-0.48,0.62)	<0.001	0.10(-0.61,0.81)	0.046	**-0.64 (-1.17,-0.11)**	0.56	-0.83 (-1.85,0.20)*	—
PND60-PND180	0.04(-0.23,0.31)	<0.001	0.20(-0.14,0.55)	<0.001	-0.17 (-0.36,0.02)	0.093	**-1.60 (-2.40,-0.79)**	<0.001
>PND180	0.31(-0.01,0.63)	0.154	1.18 (-0.76,3.12)*	0.007	-0.26 (-0.69,0.16)	0.224	—	—
Total	0.08(-0.13,0.29)	<0.001	0.25(-0.05,0.26)	<0.001	**-0.23 (-0.39,-0.06)**	0.099	**-1.49(-2.22,-0.75)**	<0.001
**Prostate weight**								
<PND60	1.09(0.42,1.76)*	—	1.43(0.73,2.12)*	—	—	—	—	—
PND60-PND180	**0.43(0.17,0.68)**	<0.001	**0.55(0.20,0.90)**	0.003	**0.33(0.16,0.51)**	0.101	**-4.21 (-5.97,2.44)**	<0.001
>PND180	**0.46(0.16,0.76)**	0.812	—	—	—	—	—	—
Total	**0.46(0.26,0.67)**	0.001	**0.63(0.29,0.98)**	0.001	0.33(0.16,0.51)	0.101	**-4.21 (-5.97,2.44)**	<0.001
**Testis weight**								
<PND60	**0.40(0.06,0.74)**	0.232	0.87(-0.28,2.02)	0.024	0.26 (-0.26,0.78)*	0.446	-1.18(-2.25,-0.10)*	—
PND60-PND180	0.03(-0.14,0.19)	<0.001	-0.09(-0.31,0.13)	0.063	0.01 (-0.21,0.23)	0.06	**-2.03(-2.78,-1.27)**	<0.001
>PND180	0.26(-0.72,1.25)	0.007	—	—	—	—	—	—
Total	0.12(-0.04,0.28)	<0.001	0.02(-0.24,0.27)	0.003	0.04 (-0.16,0.24)	0.087	**-1.92(-2.61,-1.23)**	<0.001
**Epididymis weight**								
<PND60	**0.70(0.08,1.33)**	0.024	-0.07(-0.59,0.73)	0.349	-0.40 (-1.03,0.22)	0.467	1.03 (-0.02,2.08)	—
PND60-PND180	-0.25(-0.61,0.12)	<0.001	-0.15(-0.32,0.02)	0.166	-0.13 (-0.39,0.13)	0.001	**-2.61 (-3.92,-1.30)**	<0.001
>PND180	0.62(0.31,0.93)	0.402	—	—	—	—	—	—
Total	-0.02(-0.33,0.28)	<0.001	-0.14(-0.30,0.03)	0.214	-0.15 (-0.40,0.09)	0.001	**-2.16 (-3.47,-0.86)**	<0.001
**Seminal vesicle weight**								
<PND60	**2.04(0.39,3.68)**	<0.001	2.57(1.20,3.93)*	—	0.47 (-0.47,1.41)*	—	—	—
PND60-PND180	-0.13(-0.40,0.13)	<0.001	-0.08(-0.37,0.21)	0.014	**0.25(0.04,0.46)**	0.068	-0.94 (-2.05,0.16)	<0.001
>PND180	0.41(-0.11,0.94)	0.057	—	—	—	—	—	—
Total	0.11(-0.17,0.39)	<0.001	0.04(-0.32,0.40)	<0.001	**0.26(0.05,0.46)**	0.105	-0.94 (-2.05,0.16)	<0.001
Sperm motility								
<PND60	0.40(-0.54,1.34)	0.208	—	—	—	—	—	—
PND60-PND180	**-0.94(-1.23,-0.66)**	0.133	**-0.59(-0.85,-0.32)**	0.106	-0.11 (-0.47,0.25)	<0.001	-0.56 (-1.42,0.30)	0.001
>PND180	—	—	**—**	—	—	—	—	—
Total	**-0.72(-1.08,-0.35)**	0.004	**-0.59 (-0.85,-0.32)**	0.106	-0.11 (-0.47,0.25)	<0.001	-0.56 (-1.42,0.30)	0.001
Daily sperm production (DSP; testis)								
<PND60	—	—	—	—	—	—	—	—
PND60-PND180	**-0.38(-0.54,-0.22)**	0.271	**-0.29 (-0.50,-0.08)**	0.382	**-0.28 (-0.47,-0.09)**	0.117	**-1.90 (-3.27,-0.53)**	<0.001
>PND180	**—**	—	**—**	—	**—**	—	**—**	—
Total	**-0.38 (-0.54,-0.22)**	0.271	**-0.29 (-0.50,-0.08)**	0.382	**-0.28 (-0.47,-0.09)**	0.117	**-1.90 (-3.27,-0.53)**	<0.001
Efficiency of sperm production (testis/g)								
<PND60	**—**	—	**—**	—	—	—	—	—
PND60-PND180	**-0.48 (-0.64,-0.32)**	0.236	**-0.43(-0.69,-0.18)**	0.209	**-0.49 (-0.96,-0.02)**	<0.001	—	—
>PND180	**—**	—	**—**	—	**—**	—	—	—
Total	**-0.48 (-0.64,-0.32)**	0.027	**-0.45 (-0.66,-0.23)**	0.209	**-0.49 (-0.96,-0.02)**	<0.001	—	—
**Epididymal sperm count**								
<PND60	**-1.43 (-2.38,-0.47)**	0.276	**—**	—	**-2.17 (-3.65,-0.70)**	—	—	—
PND60-PND180	**-0.45 (-0.74,-0.17)**	<0.001	**-2.48 (-3.45,-1.51)**	<0.001	**-2.47 (-3.36,-1.57)**	<0.001	**-3.42 (-3.87,-2.97)**	0.309
>PND180	**—**	—	**—**	—	**—**	—	**—**	—
Total	**-0.54 (-0.83,-0.25)**	<0.001	**-2.48 (-3.45,-1.51)**	<0.001	**-2.43 (-3.27,-1.59)**	<0.001	**-3.42 (-3.87,-2.97)**	0.309

**Note:** Effect sizes are expressed as the SMD with 95% CIs calculated using random-effects or fixed-effects models. Form each study, the dose were divided for four ascending intervals: 0-60ug/kg/day (low); 100-600ug/kg/day (medium); 1-50mg/kg/day (high); >50mg/kg/day (Extremely high), and the timing of data collection were divided for three subgroups: <PND60 (puberty and prepuberty); PND60-PND180 (adulthood); >PND180 (middle-aged and aged) to explore the effect of different doses of BPA exposure in early life (Gestation period to postnatal day 21) on reproductive system in different periods. Positive SMDs represent an increase in the outcome measure after exposure. Negative SMDs represent a decrease in the outcome measure after exposure. Number of studies (No. comparisons) of each analysis see Supplementary Material Excel Table 3.

^a^ P is a measure of heterogeneity. P>0.1; Random-effects model was used when p value for heterogeneity test <0.1; otherwise, fixed-effects model was used

^*^ This subgroup result only have one comparison, and the reliability is very poor.

### BPA at low dose (0-60ug/kg/day)

Out of 31 studies, 27 studies have applied low-dose BPA intervention in one or more experimental groups. Regardless of the period, no significant difference was found with 52 comparisons investigating the low-dose BPA on body weight (SMD: 0.08; 95% Cl: -0.13, 0.29). For organ weight, it seem that the exposure of BPA at low dose may increase the weight of prostate at any period (especially in the period of <PND60) with a moderate heterogeneity (I^2^ = 58.2%) possibly caused by the animal model and timing of collection (Prostate weight, Total: SMD: 0.46; 95% Cl: 0.26, 0.67; <PND60: SMD: 1.09; 95% Cl: 0.42, 1.76; PND60-PND180: SMD: 0.43; 95% Cl: 0.17, 0.68; >PND180: SMD: 0.46; 95% Cl: 0.16, 0.67). However, a significantly increased testis weight was only found at the period of <PND60 (SMD: 0.40; 95% Cl: 0.06, 0.74) with low heterogeneity (I^2^ = 21.5%). Interestingly, low-dose BPA exposure exhibited a significantly increased epididymis weight at the period of PND<60 (SMD: 0.70; 95% Cl: 0.08, 1.33; I^2^ = 61.2%) and PND>180 (SMD: 0.62; 95% Cl: 0.31, 0.93; I2 = 0.0%), but reverse at the period of PND60-PND180 (SMD: -0.25; 95% Cl: -0.61, 0.12; I2 = 89.0%). Meanwhile, low-dose BPA exposure exhibited a significant positive association with seminal vesicle weight at the period of <PND60 (SMD: 2.04; 95% Cl: 0.39, 3.68; I^2^ = 84.5%), same as the conclusion of testis weight.

On the aspect of sperm parameters, the results revealed that the exposure of low-dose BPA exhibited a significantly decreased sperm motility (Total: SMD: -0.72; 95% Cl: -1.08, -0.35; <PND60: SMD: 0.40; 95% Cl: -0.54, 1.34; PND60-PND180: SMD: -0.94; 95% Cl: -1.23, -0.66) with a moderate heterogeneity (I^2^ = 61.0%). In addition, when PND60 to PND180, estimates for both daily sperm production (SMD: -0.38; 95% Cl: -0.54, -0.22; I^2^ = 18.1%) and efficiency of sperm production (SMD: -0.48; 95% Cl: -0.64, -0.32; I^2^ =21.7%) were significantly associated with low-dose BPA exposure. For epididymal sperm, the exposure of BPA at low dose may resulted in a decreased count (Total: SMD: -0.54; 95% Cl: -0.83, -0.25; I^2^ = 68.9%; <PND60: SMD: -1.43; 95% Cl: -2.38, -0.47; I^2^ = 22.2%; PND60-PND180: SMD: -0.45; 95% Cl: -0.74, -0.17; I^2^ = 69.7%).

### BPA at medium dose (100-600ug/kg/day)

In total, 100 independent comparisons in 18 studies applied medium-dose BPA intervention. As with low-dose exposure, medium-dose BPA exposure had no significant effect on body weight (SMD: 0.25; 95% Cl: -0.05, 0.26; I^2^ = 75.3%), yet had sufficient effect on prostate weight, especially at the period of <PND60 (Total: SMD: 0.63; 95% Cl: 0.28, 0.98; I^2^ = 68.7%; <PND60: SMD: 1.43; 95% Cl: 0.73, 2.12; PND60-PND180: SMD: 0.55; 95% Cl: 0.20, 0.90; I^2^ = 66.1%). For testis, epididymis, and seminal vesicle weight, there was no significant association at any period (Testis weight: SMD: 0.02; 95% Cl: -0.24, 0.27; I^2^ = 56.1%; Epididymis weight: SMD: -0.14; 95% Cl: -0.30, 0.03; I^2^ = 21.6%; Seminal vesicle weight: SMD: 0.04; 95% Cl: -0.32, 0.40; I^2^ = 71.4%).

To be noted, at the period of PND60-PND180, BPA exposure at medium dose had a significant negative association with all the sperm parameters, which include sperm motility (SMD: -0.59; 95% Cl: -0.85, -0.32; I^2^ = 47.6%), daily sperm production (SMD: -0.29; 95% Cl: -0.50, -0.08; I^2^ = 5.4%), efficiency of sperm production (SMD: -0.45; 95% Cl: -0.66, -0.23; I^2^ =30.2%) and epididymal sperm count (SMD: -2.48; 95% Cl: -3.45, -1.51; I^2^ = 94.0%).

### BPA at high dose (1-50mg/kg/day)

A total of 17 studies applied high-dose BPA intervention in one or more experimental groups. When the dose increased to a high level, the negative effect of BPA on body weight seemed to begin to show (Total: SMD: -0.23; 95% Cl: -0.39, -0.06; I^2^ = 27.8%; <PND60: SMD: -0.64; 95% Cl: -1.17, -0.11; I^2^ = 0.0%; PND60-PND180: SMD: -0.17; 95% Cl: -0.36, 0.02; I^2^ = 33.6%; >PND180: SMD: -0.26; 95% Cl: -0.69, 0.16; I^2^ = 28.2%). The prostate weight was increased exposed to high-dose BPA at the period of PND60-PND180 (SMD: 0.33; 95% Cl: 0.16, 0.51; I^2^ =37.4%). However, no significant correlation was observed between high-dose BPA exposure and testis weight (SMD: 0.04; 95% Cl: -0.16, 0.24; I^2^ = 33.7%) or epididymis weight (SMD: -0.15; 95% Cl: -0.40, 0.09; I^2^ = 60.1%). Nevertheless, high-dose BPA exposure might increase the weight of seminal vesicle weight (SMD: 0.26; 95% Cl: 0.04, 0.46; I^2^ = 42.8%).

For sperm parameters, high-dose exposure could inhibit daily sperm production (SMD: -0.28; 95% Cl: -0.47, -0.09; I^2^ = 41.1%), efficiency of sperm production (SMD: -0.49; 95% Cl: -0.96, -0.02; I^2^ = 82.7%) and epididymal sperm count (SMD: -2.43; 95% Cl: -3.27, -1.59; I^2^ = 90.5%), except for sperm motility (SMD: -0.11; 95% Cl: -0.47, 0.25; I^2^ = 71.5%).

### BPA at extremely high dose (>50mg/kg/day)

Out of 31 studies, 8 studies have applied extremely high-dose BPA intervention in one or more experimental groups. The result indicated that extremely high-dose BPA exposure was significantly associated with decreased weight of body (SMD: -1.49; 95% Cl: -2.22, -0.75; I^2^ = 82.6%) and reproductive-related organ (Prostate weight: SMD: -4.21; 95% Cl: -5.97, -2.44; I^2^ = 91.1%; Testis weight; SMD: -1.92; 95% Cl: -2.61, -1.23; I^2^ = 81.4%; Epididymis weight; SMD: -2.16; 95% Cl: -3.47, -0.86; I^2^ = 95.2%), as well as negative sperm parameters (Daily sperm production; SMD: -1.90; 95% Cl: -3.27, -0.53; I^2^ = 93.3%; Epididymal sperm count; SMD: -3.42; 95% Cl: -3.87, -2.97; I^2^ = 14.8%).

### Publication bias

Funnel plot (Supplementary Figure 1) and Egger’s test (Supplementary Table 4) were used to assess publication bias. The funnel plot indicates no significant publication bias, which were confirmed by Egger’s test, respectively.

### Sensitivity Analyses

Sensitivity analysis was utilized to detect the influence of each study by repeating the meta-analysis while omitting one study each time. The result was shown in Supplementary Table 5, and no change in direction of the association was found after sensitivity analyses.

### Confidence ratings

Owing to many items of Rob were scored with “unclear”, the initial high confidence for all outcome measures was downgraded (Table 4). By reason of varying point estimates, minimal or no overlap of confidence intervals between studies, and substantial heterogeneity (I^2^ > 75%), the confidence rating of body weight and seminal vesicle weight were downgraded for unexplained inconsistency. The confidence rating of testis weight, epididymis weight and epididymal sperm concentration were downgraded for varying point estimates and moderate heterogeneity (I^2^ > 50%), whereas the sperm motility was downgraded because of varying point estimates and minimal overlap of confidence intervals between studies. All the outcome measures were not upgraded for the factors of magnitude, dose response, residual confounding and consistency species.

**Table 4:** Quality of the evidence of the overall effects of bisphenol A on the reproductive-related outcome measures using the office of health assessment and translation confidence rating methodology (NTP 2015).

**Outcome measure**	**Body of evidence (animal studies)**	**Factors for downgrading**					**Factors for upgrading**				**Final confidence rating**
		Risk of bias	Unexplained inconsistency	Indirectness	Imprecision	Publication bias	Magnitude	Dose response	Residual confounding	Consistency species	
Body weight	Initial high confidence	↓^f^	↓↓^c^	–	–	–d	–	–e	–	–	Low
Prostate weight	Initial high confidence	↓^f^	–	–	–	–d	–	–e	–	–	Moderate
Testis weight	Initial high confidence	↓^f^	↓^h^	–	–	–d	–	–e	–	–	Low to Moderate
Epididymis weight	Initial high confidence	↓^f^	↓^h^	–	–	–d	–	–e	–	–	Low
Seminal vesicle weight	Initial high confidence	↓^f^	↓↓^c^	–	–	–d	–	–e	–	–	Low
Sperm motility	Initial high confidence	↓^f^	↓^g^	–	–	–d	–	–e	–	–	Low to Moderate
Daily sperm production (testis)	Initial high confidence	↓^f^	–	–	–	–d	–	–e	–	–	Moderate
Efficiency of sperm production	Initial high confidence	↓^f^	–	–	–	–d	–	–e	–	–	Moderate
Epididymal sperm concentration	Initial high confidence	↓^f^	↓^h^	–	–	–d	–	–e	–	–	Low to Moderate

Note: –, no concern, or not present; ↓, serious concern; ↑, sufficient to upgrade evidence.

^a^ The factors “large effect magnitude” and “residual confounding” were not assessed in this study and consequently were not used to upgrade the evidence.

^b^ Serious concern because of many “unclear” scores and a change in direction of the association after sensitivity analyses.

^c^ Serious concern because of varying point estimates, minimal or no overlap of confidence intervals between studies, and substantial heterogeneity (I2 > 75%).

^d^ No strongly suspected publication bias observed.

^e^ Indications for dose–response effects either within or across studies, but the consistency of these indications was not considered sufficient to upgrade the confidence.

^f^ Serious concern because of many “unclear” scores.

^g^ Serious concern because of varying point estimates and minimal overlap of confidence intervals between studies.

^h^ Serious concern because of varying point estimates and moderate heterogeneity (I2 > 50%).

^i^ Serious concern because of varying point estimates.

^j^ No subgroup differences were estimated across species and strains.

^k^ Body of evidence was already downgraded for unexplained inconsistency and additional downgrading for imprecision was not considered appropriate (NTP 2015).

Finally, based on this stringent confidence rating, the evidence of prostate weight, daily sperm production (testis) and efficiency of sperm production were rated as moderate; the evidence of testis weight, sperm motility and epididymal sperm concentration were rated as low-to-moderate; the evidence of body weight, epididymis weight and seminal vesicle weight were rated as low.

## DISCUSSION

As a common-used chemical with weak estrogenic properties, BPA may influence the developmental plasticity during early life, and injury the hormone-sensitive organs (prostate, testis, breast, ovary et al.) [45]. In fact, despite the endocrine interference effect, its prospective promotion on the carcinogenesis and reproductive damage is still controversial.

To our knowledge, this meta-analysis is the first study that focuses on the effect of BPA exposure (low, medium, high, extremely high) on reproductive-related outcomes in male rodents (<PND60, PND60-PND180, >PND180). In our analysis, we found that different doses of BPA early-life exposure may harm the male rodents at different periods, especially in prostate weight, sperm motility, daily sperm production, efficiency of sperm production and epididymal sperm count, which has been described above.

Our analysis on body weight showed that high (1-50mg/kg/day) and extremely high (>50mg/kg/day) dose of BPA could decrease the weight of body. However, when the dose was reduced to the level of low (0-60ug/kg/day) and medium (100-600ug/kg/day), this negative association became insignificance. Another meta-analysis conducted by Wassenaar et al. [46], estimating the effect of early-life BPA exposure on body weight, revealed that the dose of >50ug/kg/day might lead to a decreased body weight, which was not absolutely aligned with our result. We supposed that it may be related to the different BPA concentrations and selection bias.

For the prostate, its correlation with estrogen has been studied *in vivo, in vitro*, and clinically [47, 48]. Due to the limited data of enrolled studies, we only analyzed the association between BPA and prostate weight. Interestingly, the result revealed that BPA could increase prostate weight at the dose of <50mg/kg/day, yet decrease at the dose of >50mg/kg/day. This trend of bi-phasic dose responses is consistent with previous findings conducted by Huang et al. [49], which manifested that 0.01-1 nM BPA promoted cell growth, but 10-1,000 nM elicited growth inhibition. This effect may be explained by several possible mechanisms. First, relatively high-dose BPA could induce cell death for its cytotoxic effects, and then decrease the weight of prostate [50]. Second, with a different affinity of BPA and ERα or ERβ, the combination of BPA and ER (ERα and ERβ) could promote cell proliferation or inhibition [51]. Generally, ERα is considered to promote the proliferation of prostatic epithelial cells, while ERβ has an anti-proliferative effect [52]. Third, evidence showed that the interaction of BPA and androgen receptors (AR) may produce an impact on the prostate [53].

From our result, the susceptiveness of the testis, epididymis, and seminal vesicle to BPA seemed to be inferior to the prostate. But interestingly, we found that puberty and prepuberty male rodents (<PND60) may be more sensitive to low-dose BPA (0-60ug/kg/day) compared with other periods and other doses. While the specific mechanism is not clear, we speculated that it might be associated with the changes of hormones and its receptors *in vivo* [54].

The analysis of sperm parameters showed a strong correlation. Early-life BPA exposure, at extremely high level (>50mg/kg/day), is an independent factor for impaired spermatogenesis and motility at any period of life. Despite the fact that the combined result of sperm motility was not significantly changed at the dose of higher than 1mg/kg/day, the negative pooled SMD (-0.11) still showed this trend. As an AR antagonist, BPA could block the normal binding activity and its interaction between AR and endogenous androgen, and thus impaired the normal spermatogenesis [55]. Concomitantly, BPA may have a direct adverse impact on spermatogenesis by targeting Sertoli cells and interrupting the meiotic progression of germ cells [56]. Also, some experi-mental studies stated that BPA could inhibit the sperm mobility, which might be mediated by means of compromising mitochondrial functions (increase the mitochondrial ROS or reduce the high mitochondrial inner transmembrane potential) and decreasing ATP levels in spermatozoa [57, 58]. In fact, the damage to sperm parameters was observed in our pooled analysis at the dose of far below 50mg/kg/day (RfD formulated by FDA for rodents) and 4mg/kg/day (RfD formulated by EFSA for rodents), which might be considered insignificant in a single study.

For male rodents, as expected, early-life exposure to doses higher than 50mg/kg/day showed a negative association with body weight, organ weight and sperm parameters. However, when we reviewed the full data (Table 3), we found a non-monotonic association (or non-monotonic dose response curves; NMDRCs) for body weight and organ weight. The mathematical definition of NMDRCs is that the slope of curve changes from positive to negative (or vice versa) at somewhere along the range of doses examined [59]. Actually, with traditional toxicology dogma “the dose makes the poison”, people always straightly believe that there is no or little toxicity under so-called “safe” dose for most endocrine-disrupting chemical (EDC) [60]. If there is a monotonic relationship between dose and effect, the assumption that a dose below NOAEL (unobserved level of adverse reactions) is a “safe” dose seems to be reliable. Yet the NMDRCs are common in the BPA *in vitro* experiments [61], and thus we deem it flawed for the premise of high dose testing to extrapolate to low “safe” doses.

Furthermore, despite we strictly followed the guidelines of SYRCLE (specifically designed to evaluate animal studies) and got robust statistical evidence through this study, some limitations have been identified. First, most studies in our analysis have an unknown Rob for many items receiving an “unclear” rating in the assessments and methodological quality. In fact, it is common for most rodent studies. To rectify this situation, in the future, scholars could use some checklists to improve the quality, for example, the ARRIVE guidelines et al. [62]. Second and unavoidingly considerable heterogeneity existed in our study, which was not effectively reduced after subgroup analysis, indicating that the heterogeneity may be attributed to the diversity of experimental design and quality. Thus, the result should be interpreted with caution. Third, owing to the limitation of data, direct evidence of damage, such as pathology, was not analyzed. Consequently, if more experiments are present in the future, the results would be more accurate.

## CONCLUSIONS

In conclusion, the result of our meta-analysis suggested a significant negative association between early-life BPA exposure and reproductive-related outcomes, especially at the dose of >50mg/kg/day. Meanwhile, sperm parameters seem to be more sensitive to BPA - the adverse effects occurred at any dose level. Moreover, we found that the NMDRCs of BPA for organ weight and body weight, which may challenge the existing “safe dose” theory. Consequently, we believe that, with more studies focused on the effect of BPA in the future, the understanding of BPA toxicity will be more limpid.

## MATERIALS AND METHODS

This meta-analysis followed the Systematic Review Centre for Laboratory Animal Experimentation (SYRCLE) [9] and PRISMA guidelines [10].

### Search strategy

We searched PubMed, EMBASE, and Toxline databases from inception until December 2019 for relevant studies on the effects of developmental exposure to BPA on the reproductive system. The search strategy in PubMed, EMBASE and Toxline database included the following domains of Medical Subject Heading (MeSH) terms: “bisphenol A”, “reproductive”, “sperm” and “rodents”. These terms were combined with “AND” or “OR”. Comprehensive search strategies were shown in Supplementary Table 1. Besides, the reference lists of included studies and related comments were manually filtered for new studies that may be relevant.

### Study screening

Two authors (RX and ZT) independently reviewed the title and abstract of the primary selection and then conducted a full-text screening when necessary. The inclusion criteria were as follows: (1) experimental rodent study; (2) exposure to BPA; (3) have an exposure history during gestation and/or lactation; (4) complete and interested outcome indicators and (5) outcomes measured in F1 males.

The exclusion criteria were as follows: (1) did not contain a control group; (2) non-English article; (3) not a rodent study; (4) no outcomes; (5) not early-life exposure; (6) exposure to a chemical other than BPA, (7) outcomes not measured in F1 generation and (8) disunity of administration unit (for example: ug/kg and ppm)

### Data extraction

The data of bibliography (journal, year and authors), animal model (species, strain, and sex), BPA exposure (time, period, dose, and frequency), study design (number of animals, duration of follow-up, and timing of data collection), and outcome measures (body weight, prostate weight, ventral prostate (VP) weight, testis weight, epididymis weight, seminal vesicle weight, sperm motility, DSP, efficiency of sperm production, and epididymal sperm concentrations) were extracted from each study. Engauge Digitizer 4.1 software (markmitch, Boston, MA, USA) was used to extract data from figures [11].

### Risk of bias (RoB)

We used SYRCLE’s RoB tool, adapted from the Cochrane RoB tool and adjusted for aspects of bias that play a specific role in animal experiments, to assess the risk of bias in the studies we included [12]. Following the instructions of SYRCLE’s RoB tool, two authors (RX and ZT) independently assessed the risk of bias, and disagreements were resolved by discussion. This RoB tool consists of 10 items, which could evaluate 6 types of bias: selection, performance, reporting, detection, attrition and other bias [12]. Moreover, we further assessed the quality of the enrolled studies from four aspects: random, blind, conflict and fund.

### Statistical analysis

Stata 16.0 software (Stata Corporation, College Station, TX, USA) was used to perform analyses. All P-values were two-sided, and P < 0.05 was regarded as significant. The results of individual study were summarized. Considering the different scale of reported data and time of data collection, as well as the average value of vast differences, standardized mean differences (SMDs) was used to calculate the combined estimates. The estimates were calculated using fixed-effects or random-effects models according to the heterogeneity, which was reported using the Cochrane Q-test [13] and the inconsistency index value [14] (Higgins et al. 2003). Funnel plot and Egger’s test were used to judge the publication bias.

### Confidence rating

The OHAT method (NTP 2015), which is based on the Grading of Recommendations, Assessment, Development and Evaluation (GRADE) method [15], was used to rate the quality of evidence. Upon OHAT method, the initial evidence was divided into four grades (high quality, moderate quality, low quality, and very low quality) following rating the features of experiment design: a) controlled exposure; b) exposure before outcome development; c) outcome assessment on the individual level; and d) inclusion of a comparison group. Based on such evaluation, four factors could increase the confidence rating (large magnitude of effect, dose-response relationship, residual confounding, and consistency across study designs) and five factors could decrease the confidence rating (risk of bias, unexplained inconsistency for the outcomes, indirectness or reduced applicability in the results, imprecision, and publication bias).

## Supplementary Material

Supplementary Figures

Supplementary Table 1

Supplementary Table 2

Supplementary Tables 3 and 4

Supplementary Table 5
