# Supplementary material for: Early-life exposure to bisphenol A and reproductive-related outcomes in rodent models: a systematic review and meta-analysis
Source: Aging (Albany NY). 2020 Sep 30;12(18):18099–126. doi: 10.18632/aging.103620 (PMC7585097; doi:10.18632/aging.103620)
Supplement: Supplementary Figures [file aging-12-103620-s005..pdf]

SUPPLEMENTARY FIGURES

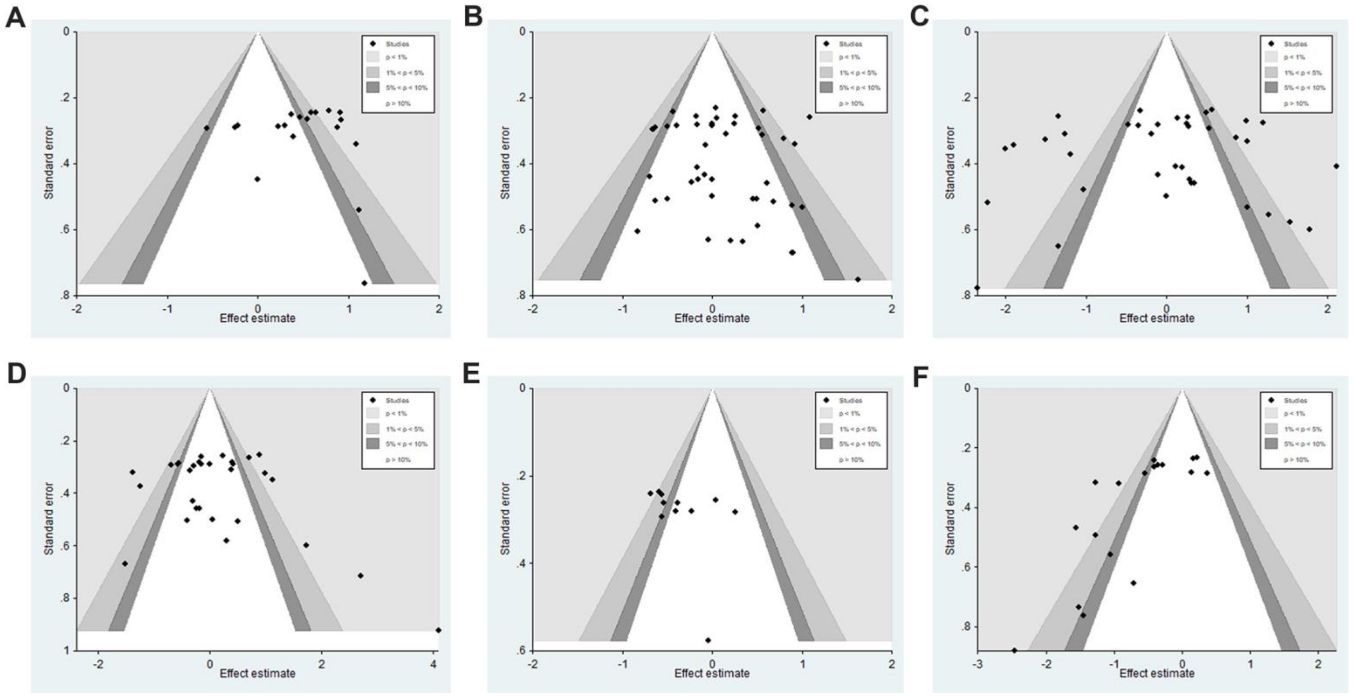

Supplementary Figure 1. Funnel plots of the analysis. Notes: (**A**) Funnel plot of prostate weight (0-60ug/kg/day); (**B**) Funnel plot of testis weight (0-60ug/kg/day);(**C**) Funnel plot of epididymis weight (0-60ug/kg/day); (**D**) Funnel plot of seminal vesicle weight (0-60ug/kg/day); (**E**) Funnel plot of daily sperm production (0-60ug/kg/day); (**F**) Funnel plot of epididymal sperm count (0-60ug/kg/day).

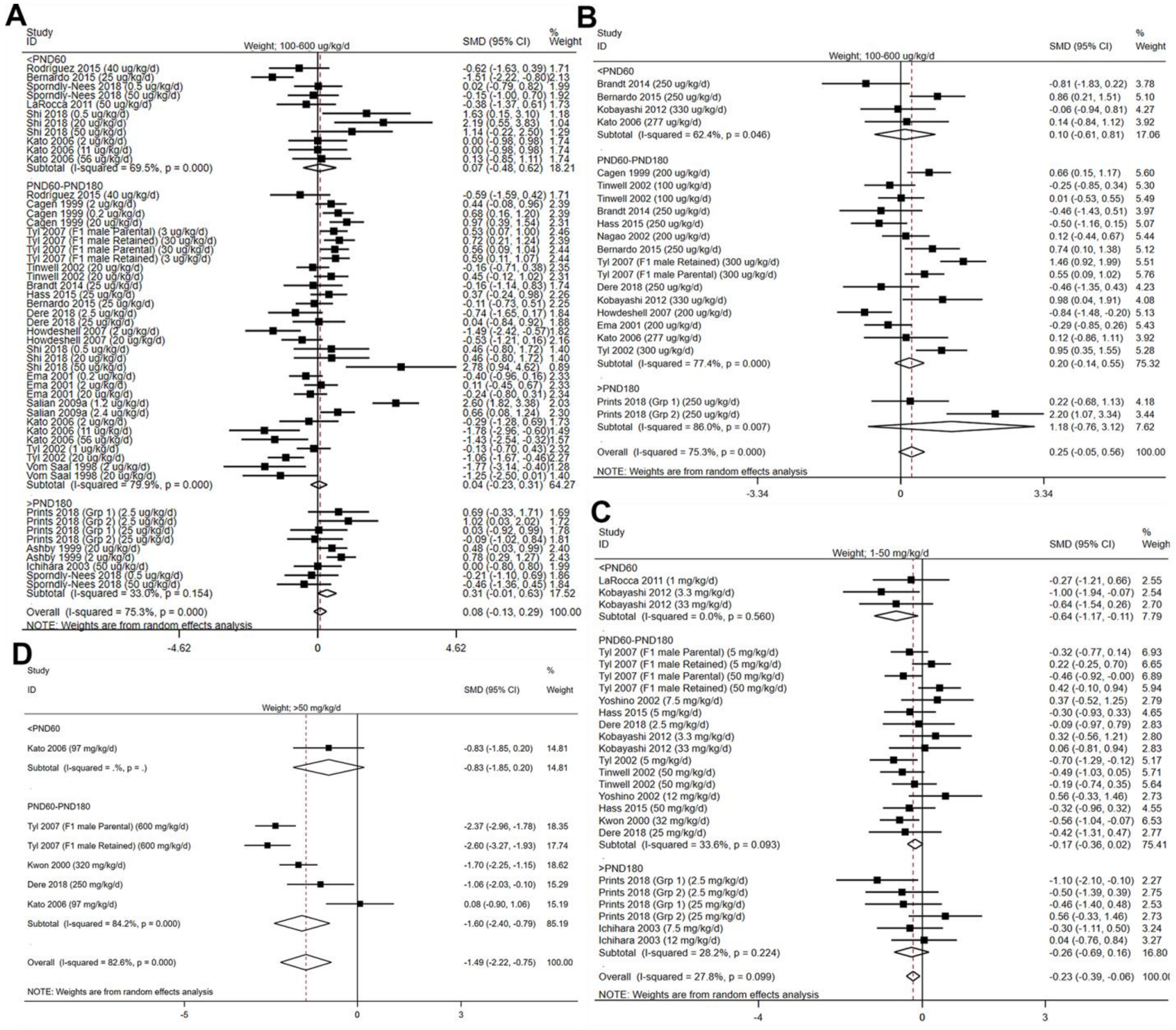

Supplementary Figure 2. Forest plots of body weight. Notes: (**A**) Forest plot of body weight (0-60ug/kg/day); (**B**), Forest plot of body weight (100-600ug/kg/day); (**C**) Forest plot of body weight (1-50mg/kg/day); (**D**), Forest plot of body weight (>50mg/kg/day).

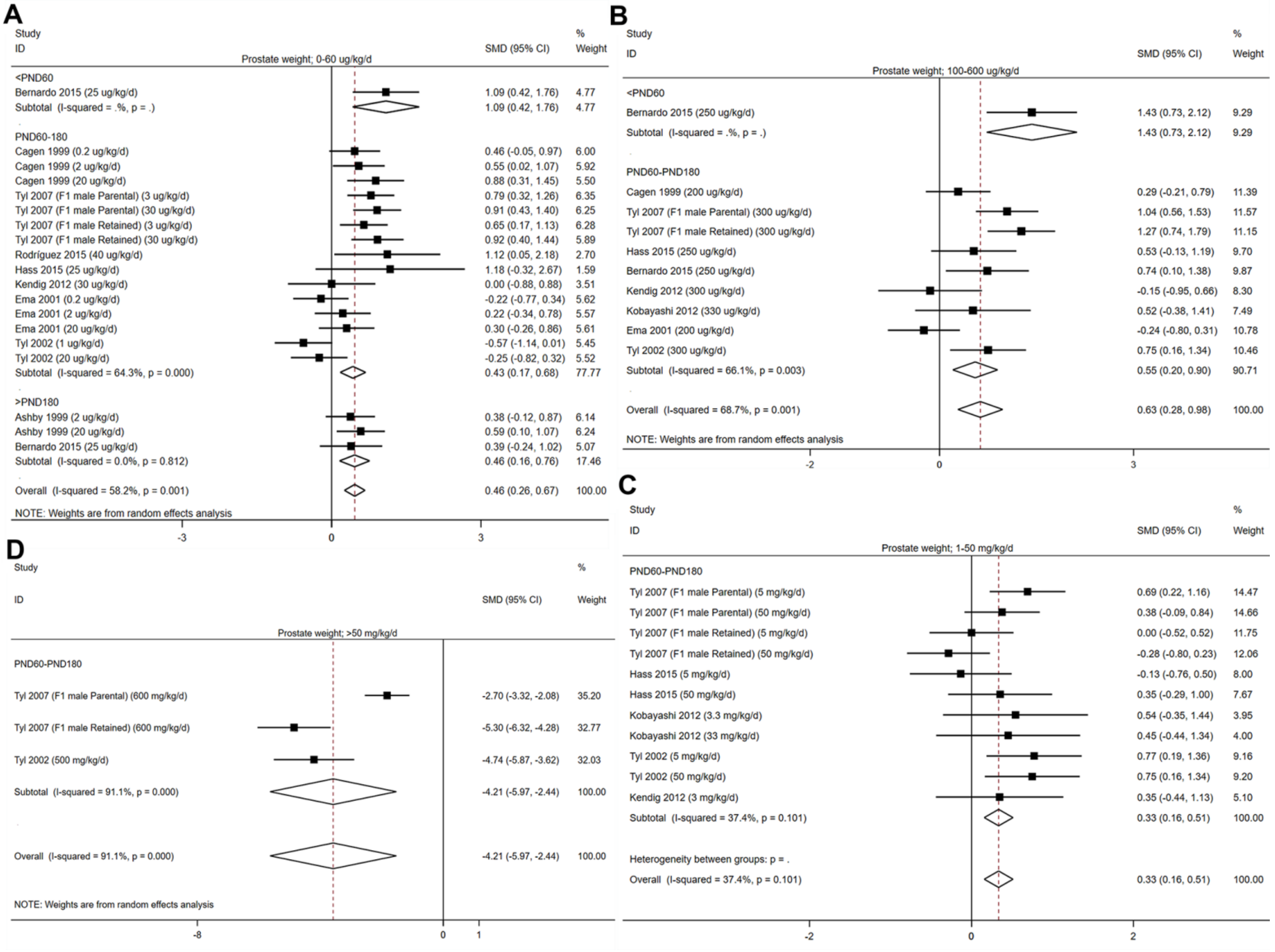

Supplementary Figure 3. Forest plots of prostate weight. Notes: (**A**) Forest plot of prostate weight (0-60ug/kg/day); (**B**) Forest plot of prostate weight (100-600ug/kg/day); (**C**) Forest plot of prostate weight (1-50mg/kg/day); (**D**) Forest plot of prostate weight (>50mg/kg/day).

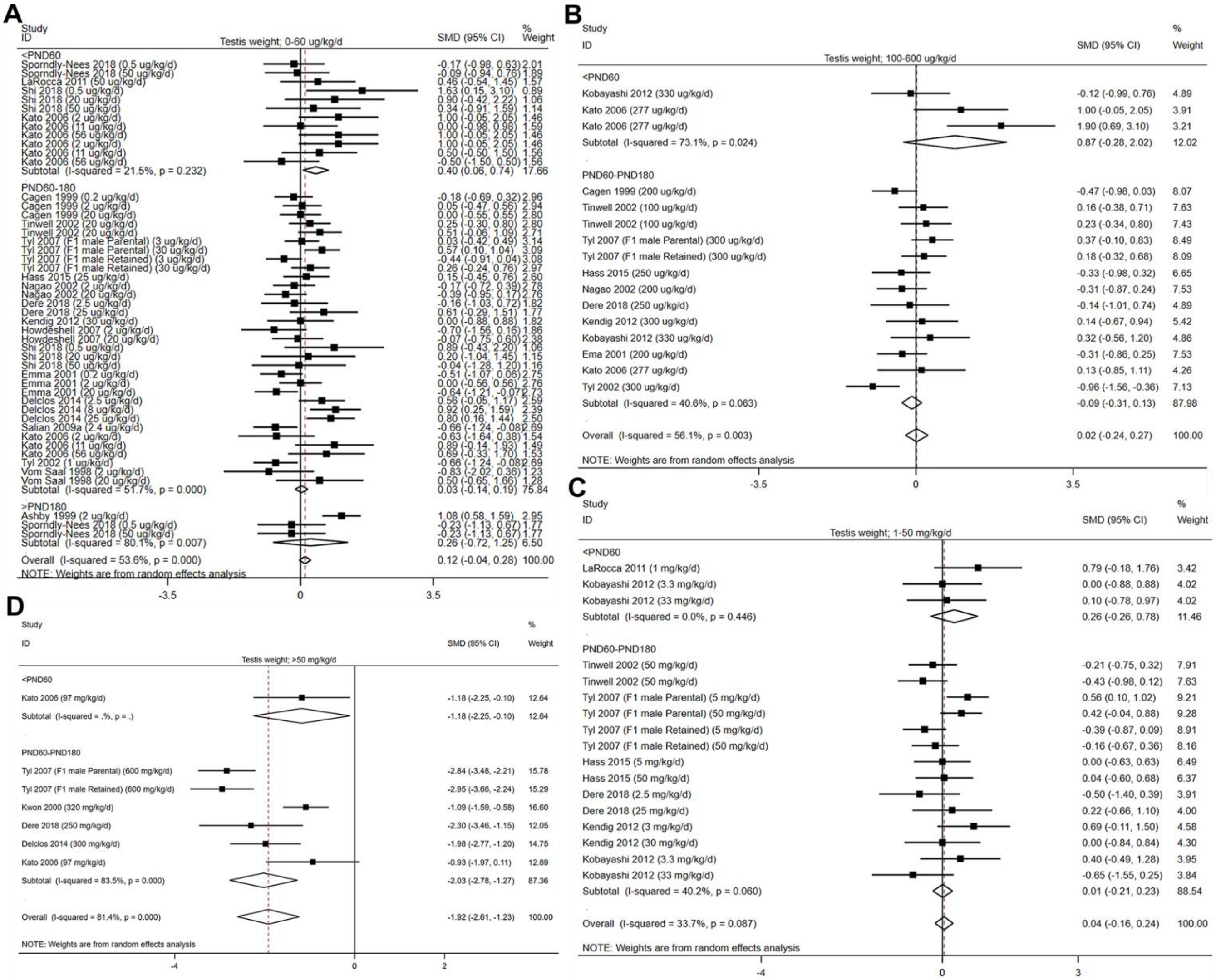

Supplementary Figure 4. Forest plots of testis weight. Notes: (**A**) Forest plot of testis weight (0-60ug/kg/day); (**B**) Forest plot of testis weight (100-600ug/kg/day); (**C**) Forest plot of testis weight (1-50mg/kg/day); (**D**) Forest plot of testis weight (>50mg/kg/day)

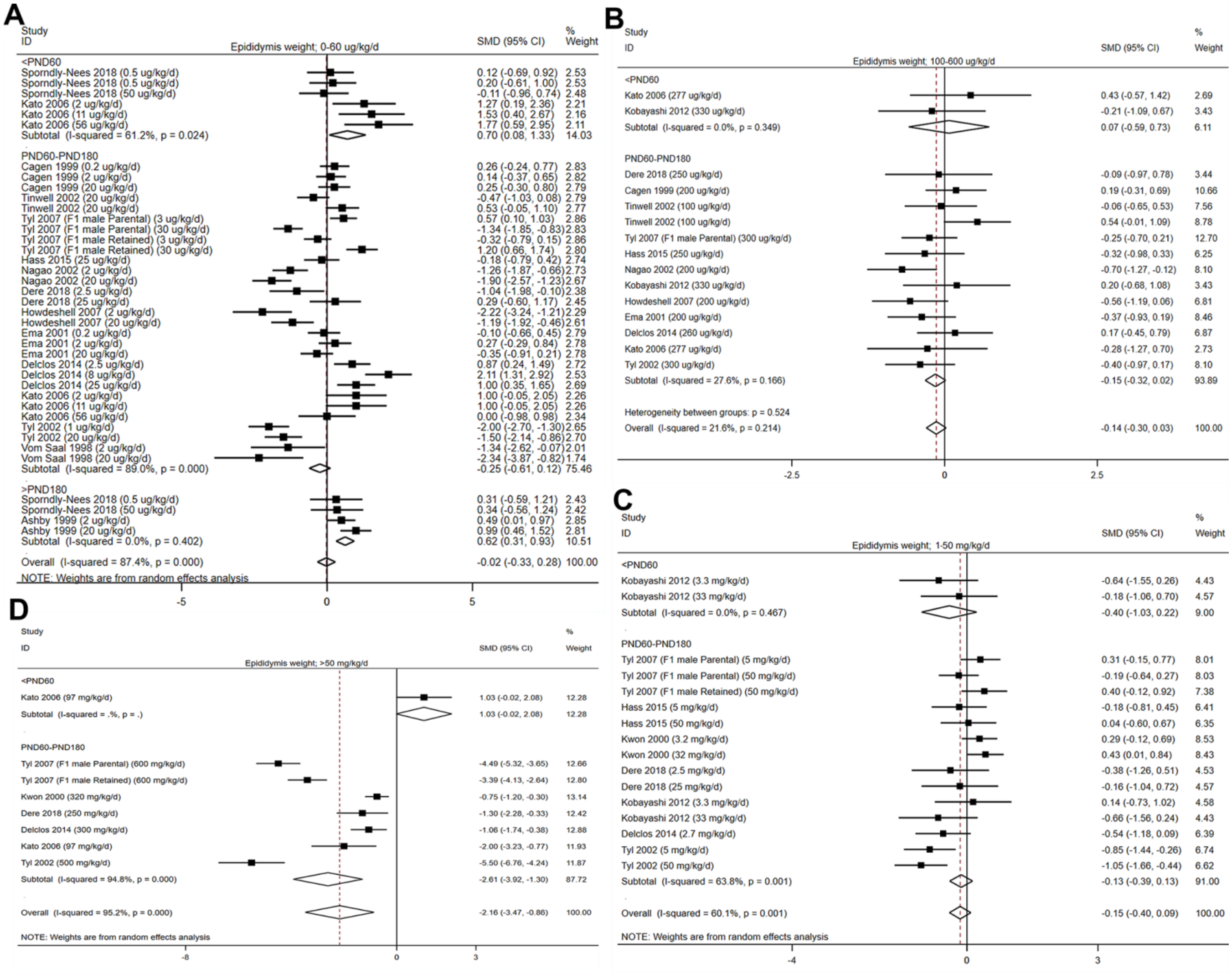

Supplementary Figure 5. Forest plots of epididymis weight. Notes: (**A**) Forest plot of epididymis weight (0-60ug/kg/day); (**B**) Forest plot of epididymis weight (100–600ug/kg/day); (**C**) Forest plot of epididymis weight (1-50mg/kg/day); (**D**) Forest plot of epididymis weight (>50mg/kg/day)

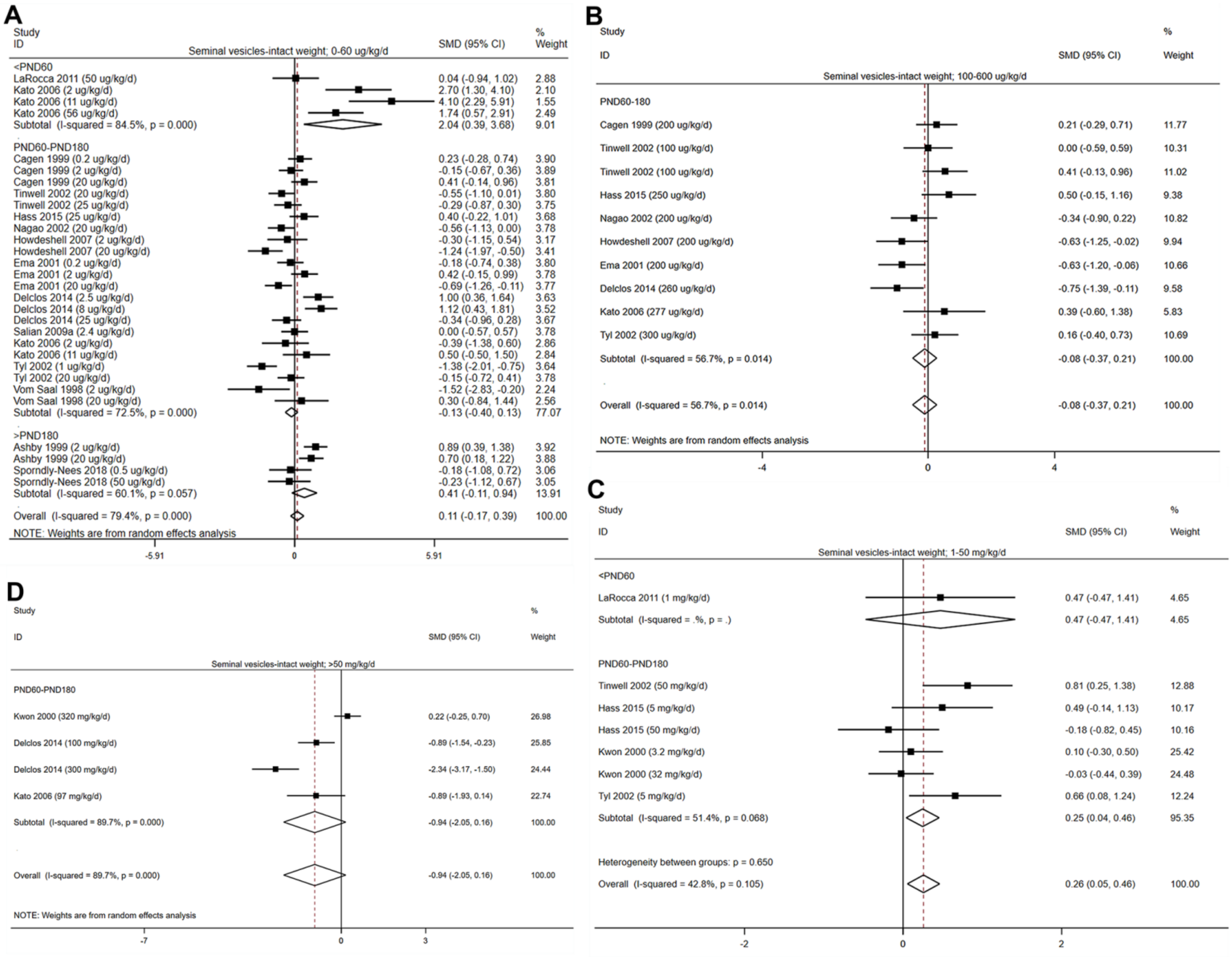

Supplementary Figure 6. Forest plots of seminal vesicle weight. Notes: (**A**) Forest plot of seminal vesicle weight (0-60ug/kg/day); (**B**) Forest plot of seminal vesicle weight (100–600ug/kg/day); (**C**) Forest plot of seminal vesicle weight (1-50mg/kg/day); (**D**) Forest plot of seminal vesicle weight (>50mg/kg/day).

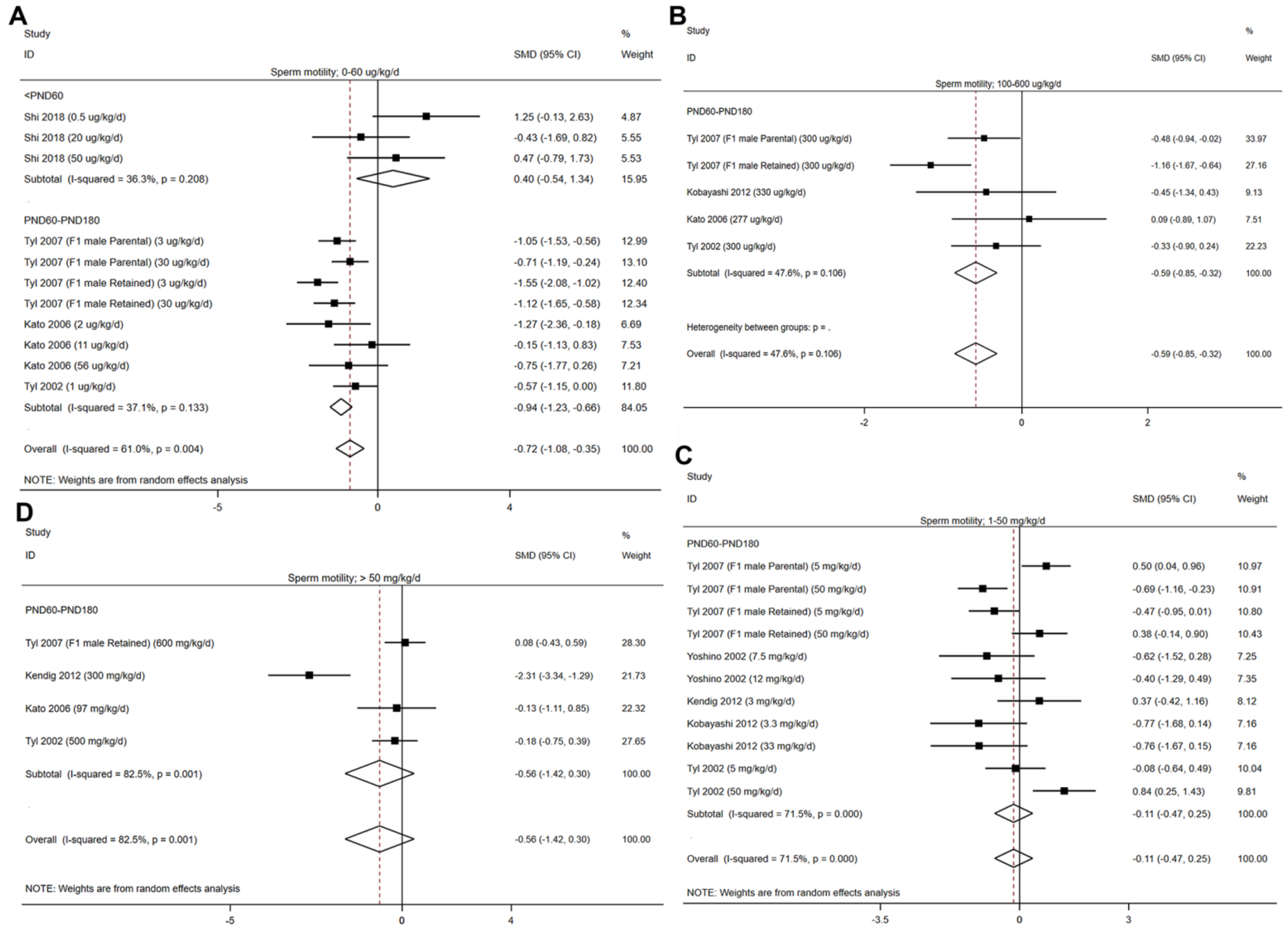

Supplementary Figure 7. Forest plots of sperm motility. Notes: (**A**) Forest plot of sperm motility (0-60ug/kg/day); (**B**) Forest plot of sperm motility (100–600ug/kg/day); (**C**) Forest plot of sperm motility (1-50mg/kg/day); (**D**) Forest plot of sperm motility (>50mg/kg/day).

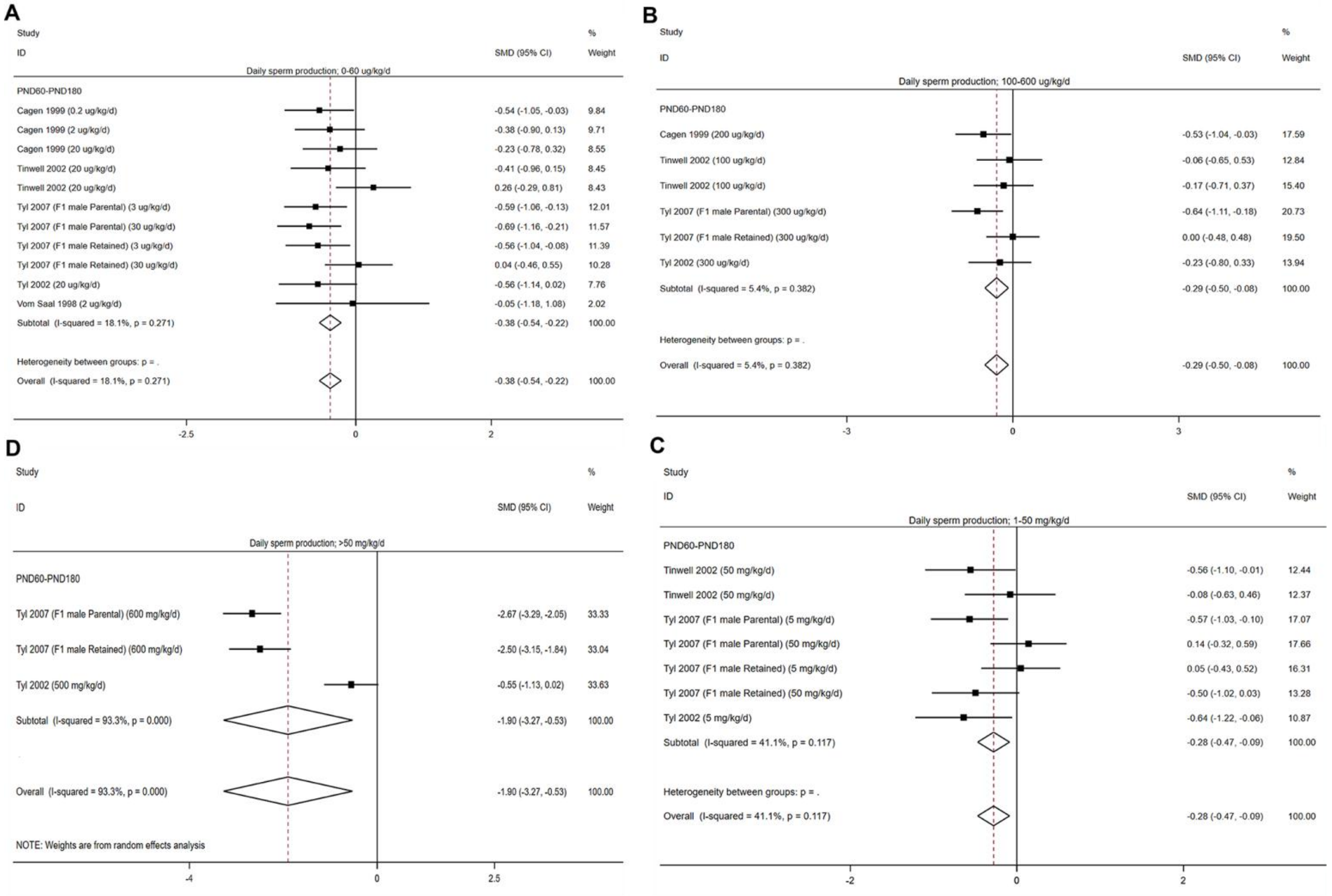

Supplementary Figure 8. Forest plots of daily sperm production. Notes: (**A**) Forest plot of daily sperm production (0-60ug/kg/day); (**B**) Forest plot of daily sperm production (100–600ug/kg/day); (**C**) Forest plot of daily sperm production (1-50mg/kg/day); (**D**) Forest plot of daily sperm production (>50mg/kg/day).

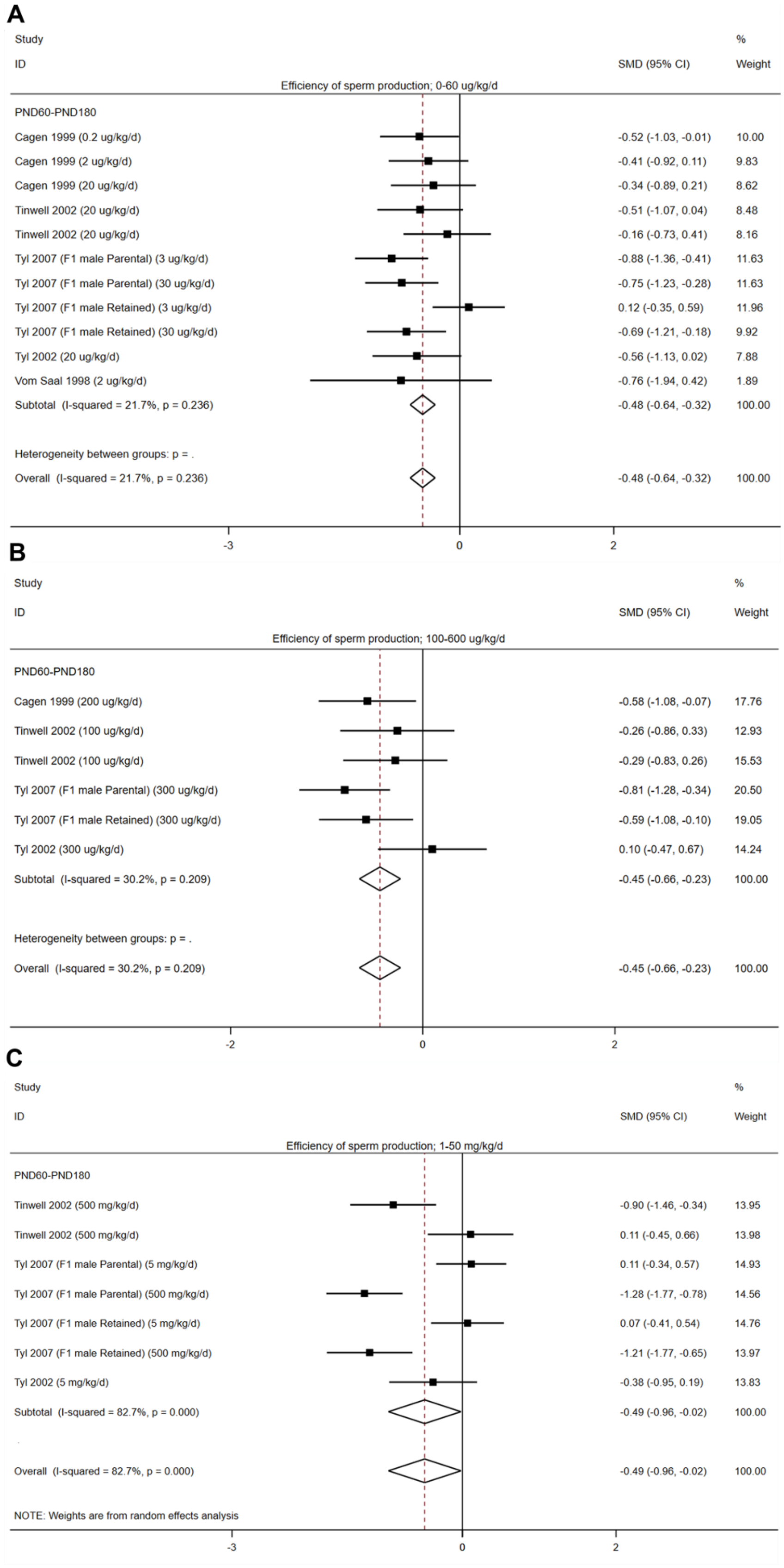

Supplementary Figure 9. Forest plots of efficiency of sperm production. Notes: (**A**) Forest plot of daily sperm production (0-60ug/kg/day); (**B**) Forest plot of daily sperm production (100–600ug/kg/day); (**C**) Forest plot of daily sperm production (1-50mg/kg/day).

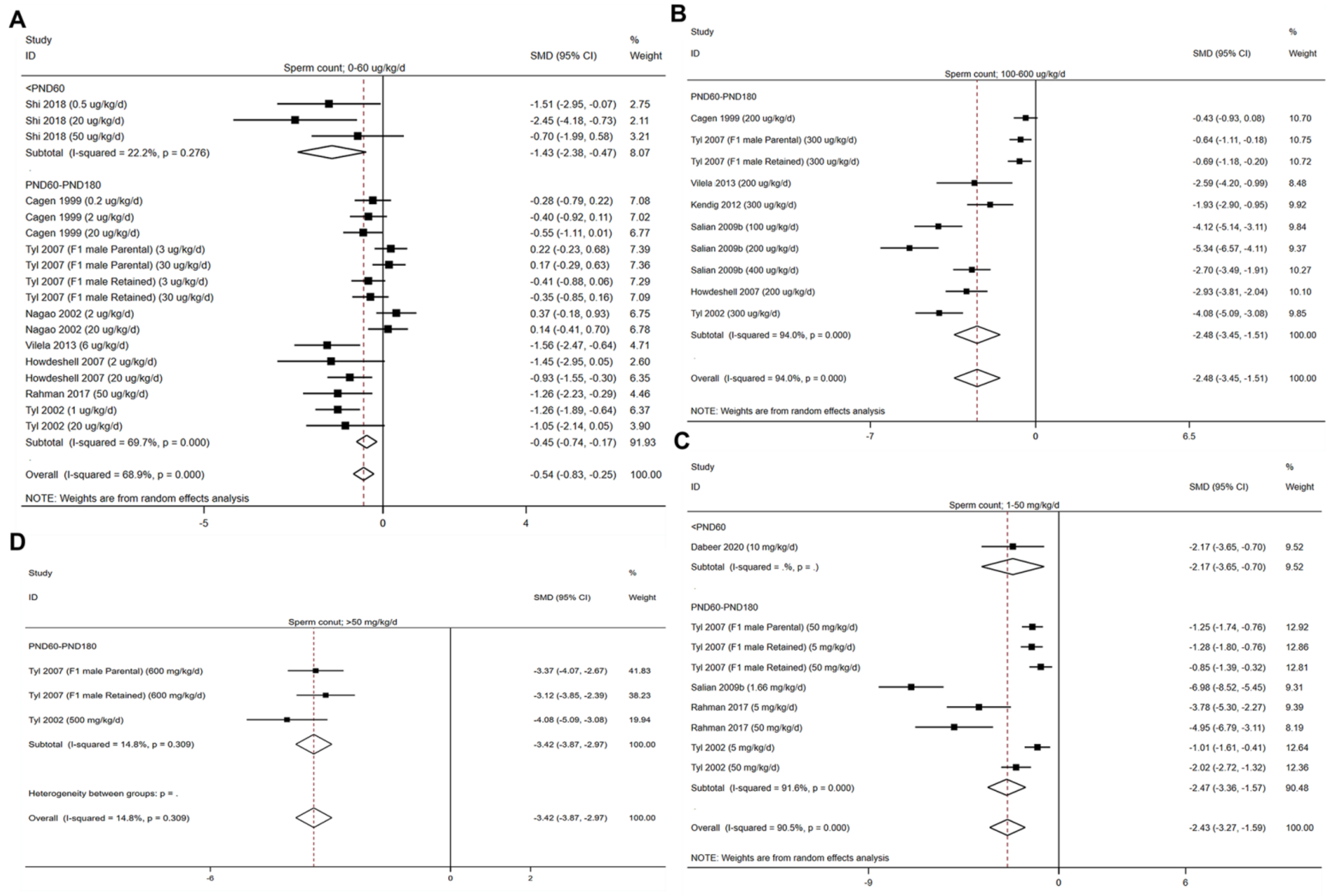

Supplementary Figure 10. Forest plots of epididymal sperm count. Notes: (**A**) Forest plot of epididymal sperm count (0-60ug/kg/day); (**B**) Forest plot of epididymal sperm count (100–600ug/kg/day); (**C**) Forest plot of epididymal sperm count (1-50mg/kg/day); (**D**) Forest plot of daily epididymal sperm count (>50mg/kg/day).
