# Supplementary material for: Early-life exposure to bisphenol A and reproductive-related outcomes in rodent models: a systematic review and meta-analysis
Source: Aging (Albany NY). 2020 Sep 30;12(18):18099–126. doi: 10.18632/aging.103620 (PMC7585097; doi:10.18632/aging.103620)
Supplement: Supplementary Tables 3 and 4 [file aging-12-103620-s004..pdf]

SUPPLEMENTARY TABLES

Supplementary Table 3. Number of studies (No. compasions) of each analysis.

Author Ref	Dose			
	0-60ug/kg/day	100-600ug/kg/day	1-50mg/kg/day	>50mg/kg/day
**Body weight**				
<PND60	6(11)	4(4)	2(3)	1(1)
PND60-PND180	15(32)	13(15)	9(16)	4(5)
>PND180	4(9)	1(2)	2(6)	—
Total	20(52)	14(21)	11(25)	4(6)
**Prostate weight**				
<PND60	1(1)	1(1)	—	—
PND60-PND180	7(15)	8(9)	5(11)	2(3)
>PND180	2(3)	—	—	—
Total	9(19)	8(10)	5(11)	2(3)
**Testis weight**				
<PND60	4(12)	2(3)	2(3)	1(1)
PND60-PND180	15(33)	11(13)	6(14)	5(6)
>PND180	2(3)	—	—	—
Total	16(48)	11(16)	7(17)	6(7)
**Epididymis weight**				
<PND60	2(6)	2(2)	1(2)	1(1)
PND60-PND180	12(28)	12(13)	7(14)	6(7)
>PND180	2(4)	—	—	—
Total	13(38)	12(15)	7(16)	6(8)
**Seminal vesicle weight**				
<PND60	2(4)	—	1(1)	—
PND60-PND180	11(22)	9(10)	4(6)	3(4)
>PND180	2(4)	—	—	—
Total	14(30)	9(10)	5(7)	3(4)
**Sperm motility**				
<PND60	1(3)	—	—	—
PND60-PND180	3(8)	4(5)	5(11)	4(4)
>PND180	—	—	—	—
Total	4(11)	4(5)	5(11)	4(4)
**Daily sperm production (DSP; testis)**				
<PND60	—	—	—	—
PND60-PND180	5(11)	4(6)	3(7)	2(3)
>PND180	1(2)	—	—	—
Total	6(13)	4(6)	3(7)	2(3)
**Efficiency of sperm production (testis/g)**				
<PND60	—	—	—	—
PND60-PND180	5(11)	4(6)	3(7)	—
>PND180	1(2)	—	—	—
Total	6(13)	4(6)	3(7)	—

**Epididymal sperm count**				
<PND60	1(3)	—	1(1)	—
PND60-PND180	7(15)	7(10)	4(8)	2(3)
>PND180	—	—	—	—
Total	8(18)	7(10)	5(9)	2(3)

Supplementary Table 4. Egger's regression test of each analysis.

**Author Ref**	**Dose**			
	**0-60ug/kg/day**	**100-600ug/kg/day**	**1-50mg/kg/day**	**>50mg/kg/day**
Body weight	0.146	0.555	0.866	0.086
Prostate weight	0.945	0.651	0.797	0.190
Testis weight	0.177	0.144	0.922	0.928
Epididymis weight	0.847	0.740	0.061	0.057
Seminal vesicle weight	0.280	0.952	0.341	0.059
Sperm motility	0.071	0.442	0.472	0.279
Daily sperm production (DSP; testis)	0.334	0.351	0.231	0.354
Efficiency of sperm production (testis/g)	0.812	0.053	0.526	——
Epididymal sperm count	0.312	0.081	0.143	0.268
